# High-Throughput Sequencing Reveals CXCR4 and IGF1 Behave Different Roles in Weightlessness Osteoporosis

**DOI:** 10.1155/2022/5719077

**Published:** 2022-04-18

**Authors:** Dong Wang, Weihang Li, Ziyi Ding, Quan Shi, Shilei Zhang, Zhuoru Zhang, Zhibin Liu, Xiaocheng Wang, Ming Yan

**Affiliations:** ^1^Department of Orthopedic Surgery, Xijing Hospital, Air Force Medical University, Xi'an, China; ^2^Department of Orthopaedics, Affiliated Hospital of Yanan University, Yanan 716000, China; ^3^Center of Clinical Aerospace Medicine, School of Aerospace Medicine, Air Force Medical University, Xi'an 710032, China; ^4^Department of Aviation Medicine, The First Affiliated Hospital, Air Force Medical University, Xi'an 710032, China

## Abstract

**Objective:**

This study is aimed at screening the differential expression profiles of mRNA under weightlessness osteoporosis through high-throughput sequencing technology, as well as investigating the pathogenesis of weightlessness osteoporosis at the molecular level especially in bone marrow mesenchymal stem cells (BMSCs).

**Methods:**

The mouse bone marrow mesenchymal stem cell line was divided into ground group and simulated microgravity (SMG) group. BMP-2 was used to induce osteogenic differentiation, and SMG group was placed into 2D-gyroscope to simulate weightless condition. Transcriptome sequencing was performed by Illumina technology, DEGs between ground and SMG group was conducted using the DEseq2 algorithm. Molecular functions and signaling pathways enriched by DEGs were then comprehensively analyzed via multiple bioinformatic approaches including but not limited to GO, KEGG, GSEA, and PPI analysis.

**Results:**

A total of 263 DEGs were identified by comparing these 2 groups, including 186 upregulated genes and 77 downregulated genes. GO analysis showed that DEGs were enriched in osteoblasts, osteoclasts cell proliferation, differentiation, and apoptosis; KEGG analysis revealed that DEGs were significantly enriched in the TNF signaling pathway and FoxO signaling pathway; the enrichment results from Reactome database displayed that DEGs were mainly involved in the transcription of Hoxb3 gene, RUNX1 recruitment KMT2A gene, and activation of Hoxa2 chromatin signaling pathway. The four genes, IL6, CXCR4, IGF1, and PLOD2, were identified as hub genes for subsequent analysis.

**Conclusions:**

This study elucidated the significance of 10 hub genes in the development of weightlessness osteoporosis. In addition, the results of this study provide a theoretical basis and novel ideas for the subsequent research of the pathogenesis and clinical treatment of weightlessness osteoporosis.

## 1. Introduction

Osteoporosis, a systemic degenerative bone disease, is characterized by systemic bone loss and hypoplasia; reduced bone density, thinning, and fracture of osteophytic bone trabeculae; and increased risk of fracture [1]. At present, antiosteoporosis drugs are clinically classified into two main categories: bone resorption inhibiting drugs and bone formation promoting drugs [2], which have achieved good results in clinical practice. However, some studies have reported [3] that astronauts suffered from weightlessness osteoporosis due to the lack of physiological mechanical load stimulation under special weightless environment of space, thus, it is almost difficult for the commonly used antiosteoporosis drugs to alleviate the progression of osteoporosis in astronauts [4], which seriously threatens the physical and mental health of astronauts and the smooth execution of flight missions. These reasons are mainly attributed to the lack of exploration in the pathogenesis and treatment of weightlessness osteoporosis.

Bone marrow mesenchymal stem cells (BMSCs) are a class of adult stem cells with self-renewal and multidirectional differentiation potential [5], which can differentiate into a host of cells including osteoblasts, such as adipocytes, chondroblasts, muscle cells, and nerve cells. Moreover, it plays an important regulatory role in maintaining normal physiological activities of the body. Studies have shown that BMSCs are important regulators in maintaining the content of osteocytes and myoblasts in vivo. Relevant scholars [6, 7] found that the differentiation of BMSCs into osteoblasts was inhibited, and adipocyte differentiation was promoted under weightlessness, which can lead to the symptoms of muscular system atrophy and osteoporosis. Therefore, studying the mechanism of BMSCs and the expression of related target genes under weightlessness environment has become the key to the prevention of weightlessness osteoporosis.

Spaceflight data [8] manifested that weightlessness-induced bone mineral density decreases at an average rate of 1.0% ~1.6% per month, which were 10~15 times faster than the rate of bone loss in postmenopausal women. Therefore, in order to ensure the astronauts successfully complete space missions in long-term spaceflight, this study is aimed at investigating the pathogenic genes and possible mechanisms of weightlessness osteoporosis, as well as providing reference for subsequent clinical drug targets.

In recent years, with the rapid development of high-throughput sequencing (HTS) technology, its characteristics of high throughput, short time, and high sensitivity make it applied to several biomedical science fields [9]. HTS technology could figure out gene sets that play important regulatory roles in various diseases. Therefore, the study firstly constructed an osteoporosis cell model under weightlessness and analyzed differentially expressed genes (DEGs) through HTS technology, followed by analysis of Gene Otology (GO) and Kyoto Encyclopedia of Genes and Genomes (KEGG) of DEGs to unveil their pathogenesis. Then, protein-protein interaction (PPI) network was constructed to explore the relationship between proteins that contribute to weightlessness osteoporosis. This will provide a strong basis for the molecular mechanism in the occurrence and development of weightlessness osteoporosis and potential drug therapy targets. Based on the findings, this study would provide effective means for early detection, diagnosis, and treatment of weightlessness osteoporosis in astronauts and make corresponding contributions to the aerospace industry ultimately.

## 2. Materials and Methods

### 2.1. Cell Culture and Experimental Conditions

**Figure 1:**
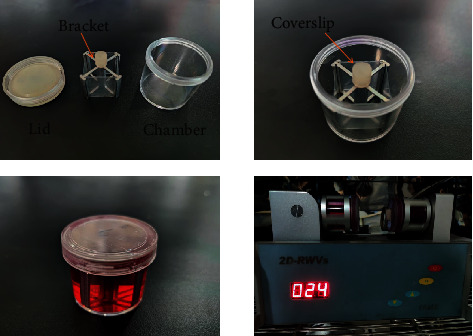
The structure of 2D-clinostat and simulated microgravity device.

Bone marrow mesenchymal stem cells (BMSCs, C3H10T1/2) were purchased from Procell Life Science & Technology Co., Ltd. (PLS, CHN) and routinely incubated in high-glucose Dulbecco's modified Eagle's medium (DMEM, Procell, CHN) containing 10% head-inactivated fetal bovine serum (FBS, Gemini, USA) at 37°C and 5% CO_2_ saturated humidity incubator. On the day before simulated weightlessness, the cells were inoculated on 2.55 cm × 2.15 cm coverslips at a density of 1 × 10^5^ pcs/piece and incubated overnight in six-well plates. After the cells reached 30% density on the coverslips, the coverslips of inoculated cells were inserted into the slot of the stainless steel bracket of the rotator and placed into the preheated rotary chamber filled with high-sugar DMEM medium containing 10%FBS, and osteogenic differentiation solution BMP-2 was added for osteogenic induction. After the bubbles were drained, the slides were placed on the rotating arm of a 2D-clinostat (2D-RWV, Rotating Wall Vessel, manufactured by China Astronaut Research and Training Center) [10]. The cells were rotated at 24 r/min for 48 h in the cell incubator. The ground group was placed next to the gyroscope and incubated for the same time. After simulated weightlessness, the cells were collected into a centrifuge tube for subsequent RNA extraction. The structure of 2D-clinostat and simulated microgravity device is shown in Figure 1.

### 2.2. Illumina High-Throughput Sequencing Database

Trizol kit (Invitrogen, Carlsbad, CA, USA) was utilized to extract total RNA from ground group and SMG group cell samples, respectively. Agilent 2100 biological analyzer was used to evaluate the quality of the extracted total RNA. After enrichment of eukaryotic mRNA with polyA tails by magnetic beads with Oligo (dT), the mRNA was interrupted by buffer. The fragmented mRNA was used as a template and random oligonucleotides as primers to synthesize the first strand of cDNA in the M-MuLV reverse transcriptase system, followed by degradation of the RNA strand with RNaseH and synthesis of the second strand of cDNA with dNTPs under the DNA polymerase I system. The purified double-stranded cDNAs were end-repaired, A-tailed, and connected to sequencing connectors, the cDNAs of about 200 bp were screened by AMPure XP beads, PCR amplification was performed, and the PCR products were purified again with AMPure XP Beads. Finally, the library was obtained and amplified and sequenced with Illumina Novaseq6000 sequencer.

### 2.3. Quality Control of Gene Expression Matrix

The generated matrices were analyzed for gene expression values to clarify the differences between groups and whether they were suitable for subsequent analysis. Principal component analysis (PCA) and correlation heat map were utilized to show the difference between these 2 groups. The genes obtained by sequencing of the two groups were conducted by Venn diagram analysis for subsequent analysis. Additionally, the violin diagram of expression values of 6 samples was visualized by *R*, which displayed the data density within each sample.

### 2.4. Screening of DEGs in Weightlessness Osteoporosis

DEseq2 algorithm was utilized to analyze the significant difference between ground group and SMG group cell samples. The screening threshold of DEGs was defined as |FC (fold change)| > 1.5 and *P* < 0.05, which was considered as statistically significant difference. The samples and genes were further performed for clustering analysis, and the heat map of DEGs was drawn by R (“pheatmap” package) to display the expression magnitude and change pattern of DEGs in each group.

### 2.5. Function and Signal Pathway Enrichment Analysis of DEGs in Weightlessness Osteoporosis

DEGs were uploaded into DAVID (http://david.abcc.ncifcrf.gov/, gene functional annotation online database tools) [11] and *R* to perform GO (Gene Ontology) and KEGG (Kyoto Encyclopedia of Genes and Genomes) analysis, and Reactome database enrichment analysis and GSEA analysis were further conducted to explore the biological significance of DEGs at the molecular level. GO is an international standard classification system for gene functions, which mainly includes cellular component (CC), molecular function (MF), and biological process (BP). KEGG is the main public database of signaling pathways, allowing information on signaling pathways related to DEGs. The Reactome database covers the reactions and biological signaling pathways of some species, more significantly enriched pathways could be found in DEGs. Gene Set Enrichment Analysis (GSEA) further explores the dominant cellular signaling pathways of DEGs.

### 2.6. Protein-Protein Interaction (PPI) Network Analysis

The STRING is a database for analyzing the interaction relationship between proteins expressed by different genes. The selected common DEGs were used to construct the interaction network using STRING database, and the obtained gene networks were imported into Cytoscape 3.8.0 software for further analysis, and then the most closely interactive subnetworks were further screened based on MCC algorithm. Studies [12] have shown that MCC algorithm is aimed at identifying the most relevant key targets and subnetworks from the complex and huge interaction network, which is one of the most effective methods to identify target genes. This contributes to study the molecular mechanisms of diseases and new targets of drugs from a more comprehensive perspective.

### 2.7. Western Blotting Assay

Western blotting assay was primarily used to detect the expression levels of related proteins. BMSCs were inoculated in T25 culture flasks, and after inducing osteogenic differentiation, the samples of SMG group were subjected to simulated weightless experiment. Then, total proteins were extracted from BMSCs of the two groups separately using phenylmethylsulfonyl fluoride (PMSF) cell lysis buffer. The Pierce BCA protein assay kit (Beyotime Biotechnology, China) was utilized to determine the protein standard curves and the protein concentrations of both samples. Then, the protein samples were mixed with buffer solution and boiled at 100°C. Proteins were isolated by 10% sodium dodecyl sulphate-polyacrylamide gel electrophoresis (SDS-PAGE) and then transferred onto polyvinylidene fluoride (PVDF) membrane via semidry transfer method. The membrane was sealed at room temperature for 2 hours with 5% skim milk, TRIS buffer brine and Tween 20 (TBST) buffer; primary antibodies including CXCR4, IGF1, and GAPDH (Abcam, UK) were diluted and incubated overnight at 4°C. And the membranes were incubated by goat anti-rabbit horseradish peroxidase conjugate (Abcam, UK) at room temperature for 1 h. Finally, protein signals were detected with an enhanced chemiluminescence kit, and band density was measured using the Viber Bio imaging tool.

### 2.8. Statistical Analysis

All numerical data were expressed as mean ± standard deviation of three independent replicate experiments. The results were statistically compared using the Student's *T*-test. *P* < 0.05 was considered a statistically significant difference. All statistical analyses were conducted by SPSS19.0 software (SPSS Company, USA).

## 3. Results

### 3.1. Data Preprocessing of Expression Matrix

**Figure 2:**
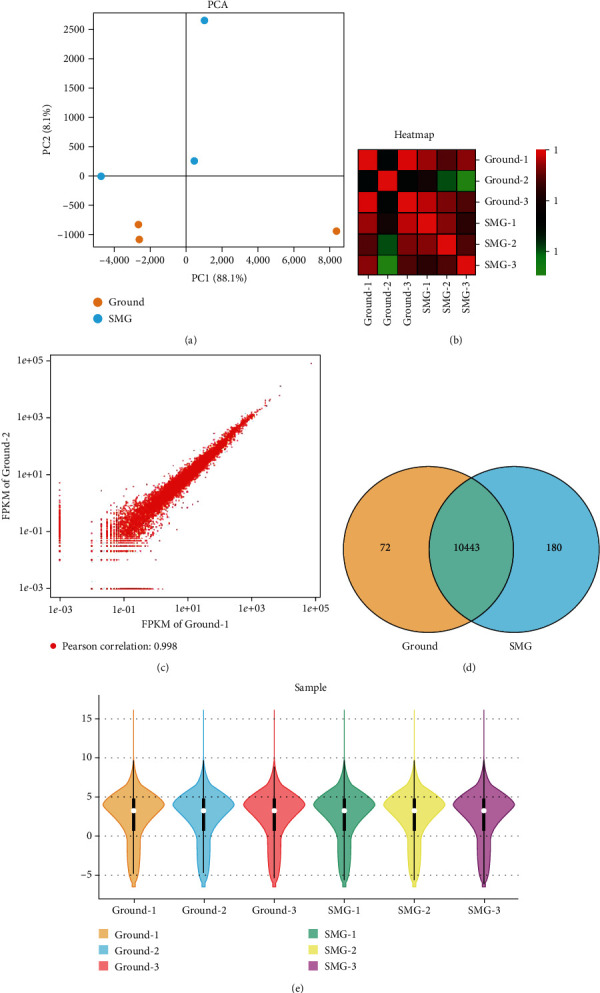
Sample relationship and data analysis. (a) Principal component analysis, (b) heatmap analysis, (c) repeatability scatter plot analysis, (d) Venn plot analysis, and (e) violin diagram of sample of ground group and SMG group.

According to the results of Illumina sequencing instrumentation, relationships between sample and the obtained data were conducted to get a comprehensive assessment. PCA analysis revealed that the ground and SMG group were distinguishable on the PC2 axis (Figure 2(a)); correlation heat map analysis showed a strong correlation between the two groups, which was valuable for subsequent analysis (Figure 2(b)); repeatability scatter plot indicated that the data distribution within the group was positively correlated (Figure 2(c)). Venn plot demonstrated that there were 10,443 genes that simultaneously correspond to both groups after sequencing (Figure 2(d)); the violin diagram displayed that the expression of six samples conformed to normal distribution (Figure 2(e)).

### 3.2. Expression Analysis of DEGs in Weightlessness Osteoporosis

**Figure 3:**
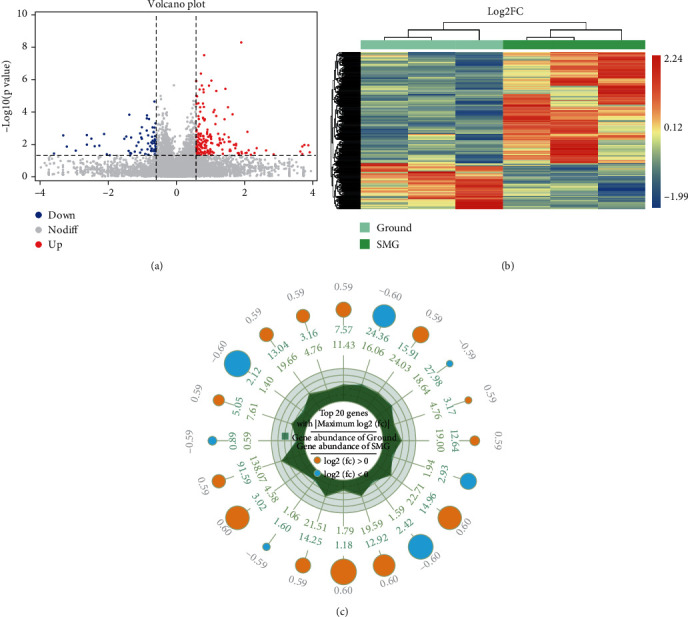
DEG screening and expression level analysis of different samples. (a) Volcano plot analysis of selected DEGs from Ground group and SMG group. (b) Heatmap of hierarchical clustering analysis of selected DEGs from ground group and SMG group. (c) Radar plot analysis of selected DEGs from ground group and SMG group.

By setting the threshold of |FC (fold change)| > 1.5 and *P* < 0.05, the sequencing results of ground and SMG groups showed that a total of 263 DEGs were detected, including 186 upregulated genes and 77 downregulated genes, and their specific gene distribution was shown in the volcano plot (Figure 3(a)), at the same time, the results of hierarchical clustering analysis demonstrated that the expression levels of common DEGs between ground group and SMG group were significantly different (Figure 3(b)). This study further exhibited the expression of the top 20 DEGs, as shown in the radar plot (Figure 3(c)). The specific coupregulated and codownregulated genes are shown in Supplementary Table 1.

### 3.3. Functional Enrichment Analysis of DEGs

**Figure 4:**
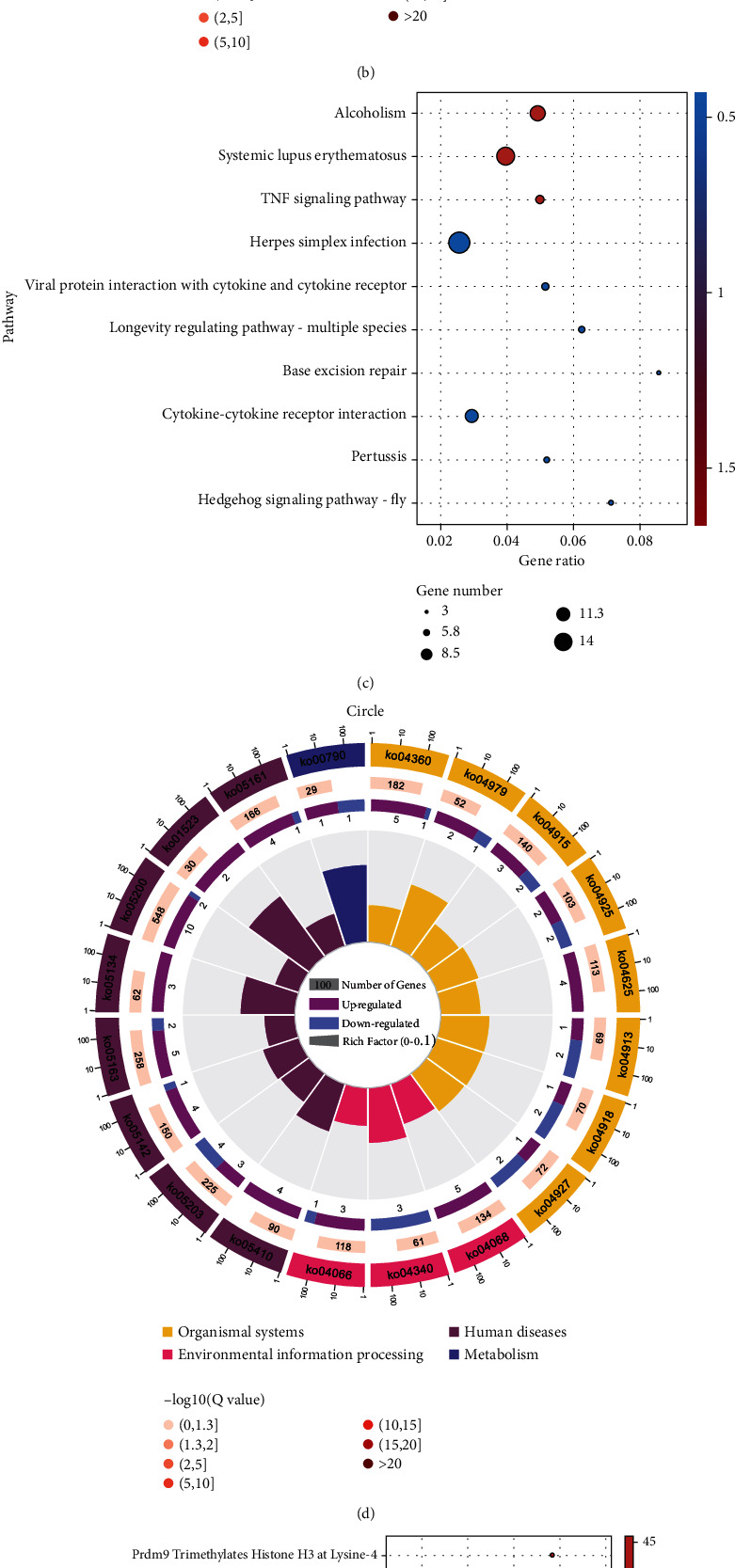
GO, KEGG, and Reactome analysis results of DEGs. (a) Bubble diagram and (b) circle diagram exhibited GO enrichment analysis results of the DEGs. (c) Bubble diagram and (d) circle diagram showed KEGG enrichment analysis results of the DEGs. (e) Bubble diagram and (f) circle diagram displayed Reactome pathway analysis results of the DEGs.

The screened DEGs were uploaded into the DAVID database and *R* for GO functional enrichment analysis in weightlessness osteoporosis. Results of GO elucidated that the upregulated DEGs in BP were mainly involved in the negative regulation of multicellular biological processes, ribonucleic acid biosynthesis process, and cell-cell adhesion; in MF, they were chiefly associated with the regulation of cell differentiation, antioxidant activity, and regulation of cellular value-added; in CC, they were primarily enriched in the extracellular matrix containing collagen, synapses, and Golgi vesicles. Downregulated DEGs multicellular biological processes, MSC migration, and mitotic cell cycle phase transition were mainly involved in BP; regulation of nucleobase-containing compound metabolism and ATPase activator activity was principally associated with MF; and supramolecular complexes, intracellular membrane-bound organelles, and chromatin were mainly enriched in CC. Furthermore, bubble plots displayed the top 10 terms with the most significant differences from the results of GO, as shown in Figure 4(a), and the next 20 terms with significant differences were shown in chord plots (Figure 4(b)). In addition, the enrichment results of GO with all specific thresholds *P* < 0.05 are shown in supplementary Table 2.

### 3.4. Signaling Pathway Enrichment Analysis of DEGs

**Figure 5:**
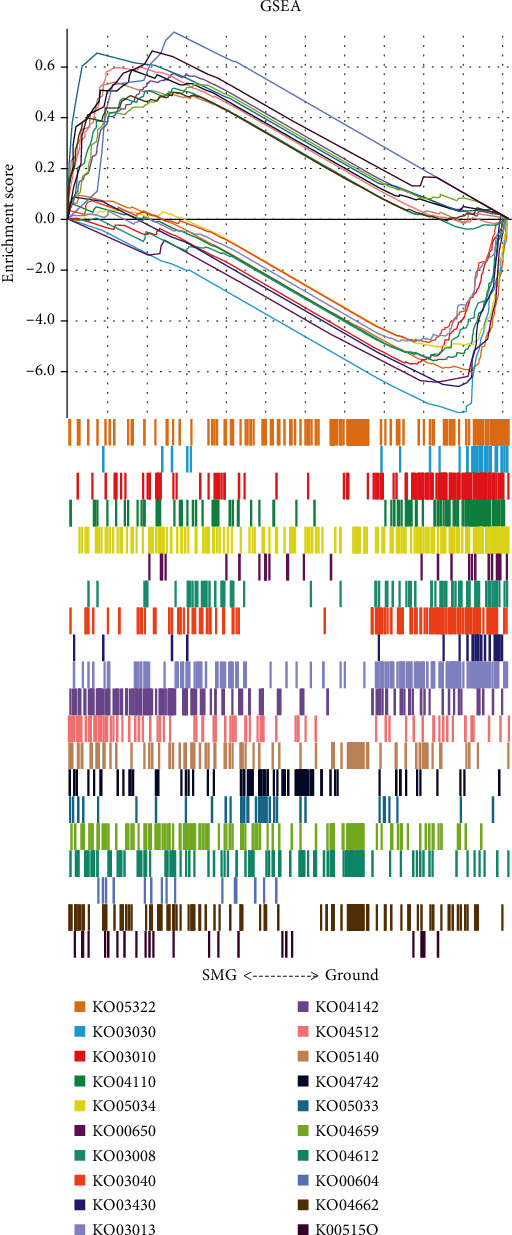
GSEA analysis results of DEGs.

The screened DEGs were uploaded into the DAVID database and *R* for KEGG, Reactome, and GSEA signal pathway enrichment analysis in weightlessness osteoporosis. Enrichment analysis of KEGG revealed that DEGs was primarily involved in the interaction between cytokines, TNF signaling pathway, and FoxO signaling pathway. The top 10 signaling pathways with the strongest correlation were selected and displayed as bubble diagrams (Figure 4(c)), and the next 20 signaling pathways with strong correlation were selected to draw chord plot, as shown in Figure 4(d). Further pathway enrichment analysis was also performed in the Reactome database to explore the specific signaling pathways. Moreover, enrichment analysis of Reactome signal pathway further suggested that DEGs were mainly enriched in the transcription of Hoxb3 gene, RUNX1 recruitment of KMT2A gene, and activation of Hoxa2 chromatin signaling pathways. The top 10 most relevant signaling pathways were displayed as bubble diagrams (Figure 4(e)), and the next 20 signaling pathways with the most correlation were shown in chord diagrams (Figure 4(f)). In addition, the enrichment results of KEGG and Reactome with all specific thresholds *P* < 0.05 are shown in supplementary Tables 3 and 4, respectively. GSEA analysis in this study further indicated that the upregulated pathways primarily included ribosomal biogenesis, gene replication, and RNA transport in eukaryotes, while downregulated pathways mainly include ECM receptor interaction, Th17 cell differentiation, and B cell receptor signaling pathway (Figure 5).

### 3.5. Protein-Protein Interaction Network Analysis (PPI)

**Figure 6:**
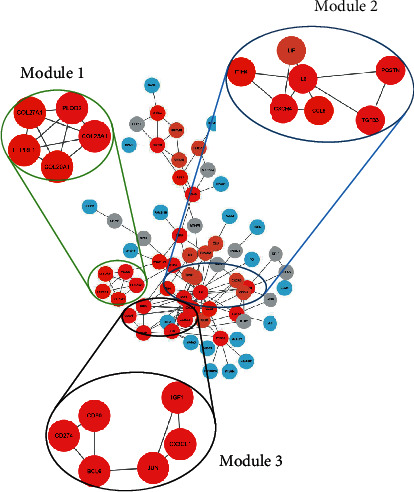
Interaction between DEGs in weightless osteoporosis. PPI network analysis of DEGs as well as the top 3 modules from PPI network. Every edge represented the interaction between two genes.

After analyzing the functions and aberrant signaling pathways, the common DEGs were then applied to construct PPI networks using the STRING database, and the generated gene networks were imported into Cytoscape 3.8.0 software for further analysis. The MCC algorithm was utilized to identify subnetworks with the closest interactions within gene networks, and totally, 3 subnetworks were generated. The top 10 genes calculated by MCC values were as follows: IL6, CXCR4, IGF1, JUN, CD80, CD274, BCL6, CX3CL1, COL23A1, and PLOD2. The detailed results were shown in Figure 6, and the darker color indicated the more significant role of the genes in the progression of weightlessness osteoporosis.

### 3.6. Validation Results of Western Blotting

**Figure 7:**
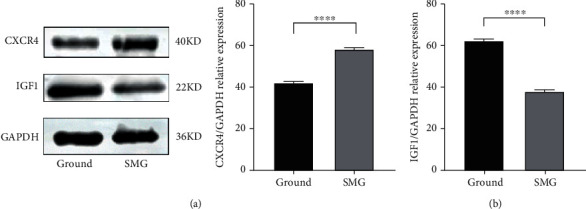
Validation of hub genes under weightlessness osteoporosis. Western blotting analysis of CXCR4 (a) and IGF1 (b) protein expression level in BMSCs after 48 h simulated microgravity. Data are presented as mean ± SD.

The expression of CXCR4 and IGF1 was detected by Western blotting analysis. As shown in Figures 7(a) and 7(b), CXCR4 was highly expressed in the weightless environment, while IGF1 was lowly expressed. This is consistent with the findings of this study.

## 4. Discussion

Osteoporosis, currently recognized as the most prevalent systemic degenerative bone disease around the world, is mainly caused by the functional imbalance between osteoblasts and osteoclasts. It is mainly divided into primary and secondary osteoporosis, and secondary osteoporosis [13] primarily includes endocrine metabolic diseases that affect bone metabolism, drug-related, disuse, and other osteoporosis with clear etiology. Among which weightlessness osteoporosis caused by space flight is a special kind of disuse osteoporosis [14]. Some studies have shown [15] that the dominant reason is bone loss due to reduced skeletal loading and mechanical forces under weightlessness. Some scholars have also found [16] that weightlessness osteoporosis is associated with the redistribution of body fluids throughout the body after mankind enter the space. Nevertheless, there are few researches focusing on the molecular targets that contribute to the occurrence of weightlessness osteoporosis. In order to find effective potential therapeutic targets, it is necessary to further explore the pathogenesis of weightlessness osteoporosis.

In recent years, with the rapid development of HTS technology together with bioinformatics, it has been widely applied to study the occurrence and development of diseases and further analyze the disease-related causative genes and their biological significance [17–19]. Besides, the novel drug screening based on hub genes has also been reported [20]. Thus, this study is aimed at investigating the regulatory mechanisms and primary functions of pathogenic genes in the occurrence and development of weightlessness osteoporosis, aiming at providing novel ideas for the pathogenesis of osteoporosis and subsequent therapeutic targets.

GO enrichment analysis showed that the downregulated DEGs were mainly involved in the negative regulation of biological processes, MSC migration, mitotic cell cycle phase change, and osteoclast maturation, which indicated that osteoblasts were prone to osteoporosis progression under weightless condition; enrichment analysis of KEGG signal pathway revealed that the upregulated DEGs were mainly involved in cytokine interactions, TNF signaling pathway, and FoxO signaling pathway. Previous research reported that TNF mediated the development of postmenopausal osteoporosis and osteoporosis in systemic lupus erythematosus (SLE) patients, which is consistent with the results in this study that TNF signaling pathway also mediated osteoporosis in weightlessness condition; enrichment analysis of Reactome signal pathway elucidated that DEGs were primarily involved in Hoxb3 gene transcription, RUNX1 recruitment of KMT2A gene, and Hoxa2 chromatin activation signaling pathway, suggesting that Hoxb3 and RUNX1 related signaling pathways also played a crucial role in weightlessness osteoporosis. GSEA analysis further indicated that DEGs are mainly involved in ribosome biogenesis, gene replication and RNA transport, ECM receptor interaction, Th17 cell differentiation, and B cell receptor signaling pathway in eukaryotes. These functions may have a certain impact on bone tissue, mainly including regulation of osteoblast differentiation, bone development, bone mineralization, regulation of bone resorption, intramembranous bone growth, and regulation of bone remodeling. The abovementioned functions and signaling pathways related to DEGs are the key pathogenesis that mediated weightlessness osteoporosis.

The occurrence and development of weightlessness osteoporosis are the result of the joint action of multiple genes and signaling pathways. In this study, PPI network was further constructed to identify and authenticate synergistic genes, and the top 10 genes were calculated using the MCC algorithm, namely, IL6, CXCR4, IGF1, JUN, CD80, CD274, BCL6, COL23A1, and PLOD2. Among them, IL6, CXCR4, IGF1, and PLOD2 were finally included in this study for follow-up analysis, because the relationships between these genes and weightlessness osteoporosis remained unclear, which was worth further analyzing. Therefore, these gene sets would provide new ideas for understanding the pathogenesis of weightlessness osteoporosis.

IL6 is a multibiological characteristic cytokine located on human chromosome 7p21, which is not only widely presented in various types of inflammations as a common inflammatory factor but also played important roles in multiple stages and systems of biology, such as cirrhosis [21]. Related scholars have found that IL6 could promote the formation and absorption of bone cells [22–24]. A clinical study showed [25] that IL6 mRNA was highly expressed in bone explants of patients with pyramidal fractures due to osteoporosis. It has also been found [26] that some inflammatory factors such as IL6 may upregulate the expression of RANKL in osteoblasts and accelerate RANKL signaling transduction, which directly resulted in bone destruction. Our study further confirmed that IL6 is closely associated with the progression of osteoporosis in weightlessness. CXC chemokine receptor type 4 (CXCR4) is a 7-transmembrane G-protein-coupled receptor [27], which is widely expressed in peripheral blood and other organ tissues, including hematopoietic stem cells, endothelial cells, lymphocytes, and cancer cells [28], and mainly participates in chemotaxis in the immune and hematopoietic systems [29]. Studies [30] have shown that SDF-1/CXCR4 played a significant role in the occurrence and development of bone differentiation, bone regeneration, and orthopedic diseases, such as neovascularization, BMSC migration, and cytokine secretion. In addition, SDF-1/CXCR4 could also promote BMSCs bone regeneration and differentiation through MAPK signaling pathway [31]. However, our findings revealed that CXCR4 was highly expressed in weightlessness osteoporosis, which may contradict the low expression state in normal osteoporosis. Combined with the previous finding that vascular endothelial cells tend to apoptosis in weightlessness condition via SDF-1/CXCR4 signaling pathway [32], this study deduced that the SDF-1/CXCR4 pathway mainly mediated the apoptotic process of vascular endothelial cells rather than bone differentiation under weightlessness condition. Procollagen-lysine, 2-oxoglutate 5-dioxygenase (PLOD2) is one of the members of the PLOD family. Studies [33–35] showed that PLOD2 was mediated by HIF-1*α*, TGF-*β*, and microRNA-26a/b to promote the occurrence and development of gliomas. Meanwhile, PLOD2 induced the differentiation of bone marrow stromal cells [36], and the abnormal expression of PLOD2 could result in Bruck syndrome [37], which is manifested as osteogenesis imperfection, bone fragility, and brittle fracture. This study firstly found that PLOD2 played a momentous role in mediating the progression of weightlessness osteoporosis. IGF1, namely, insulin-like growth factor I, is also a protein encoded by human gene and an important product of multiple cells through autocrine and paracrine secretion. It has been shown that IGF1 promoted metastasis of melanoma cells and also promoted migration and invasion of hepatocellular carcinoma cells through upregulating the expression of EGR1. IGF1 also played a vital anabolic role in skeletal development [38], and low expression of IGF1 could induce risk of osteoporosis fracture, which was consistent with the conclusion in this study.

## 5. Conclusions

In conclusion, finding new drug targets to prevent or slow down weightlessness osteoporosis is one of the major issues that need to be urgently addressed in spaceflight. In this study, DEGs were identified by high-throughput sequencing by weightlessness osteoporosis model, and the synergistic relationships between their DEGs were analyzed to further explore the regulatory mechanisms affecting the occurrence of weightlessness osteoporosis. IL6, CXCR4, IGF1, and PLOD2 played crucial roles in weightlessness osteoporosis, which provided a theoretical basis for the pathogenic mechanism and treatment of weightlessness osteoporosis.

## Data Availability

The raw data of Bulk-RNA sequencing used to support the findings of this study are deposited at the NCBI with the accession ID SUB11153018, which would be released on Jan 1, 2023, on NCBI due to some conflicts of interest and the importance of our experimental data. Alternatively, the same raw data could also be found at Figshare, and the URLs are as follows. Gene matrix data used to support the findings of this study are available from the corresponding author upon request. We uploaded a total of 6 files to Figshare, and the URLs are as follows: https://figshare.com/s/74eb3ca1839318a2b249https://figshare.com/s/ef0cd053ad787107410dhttps://figshare.com/s/a02ce47840e7ecef1af6https://figshare.com/s/6093aec3ae70d11e9af6https://figshare.com/s/6fe3d14cbdbd9820a3adhttps://figshare.com/s/8adf677a50441fb43b92

## Supplementary Materials

Supplementary Materials The specific coupregulated and codownregulated genes are shown in supplementary Table 1. The detailed enrichment results of GO, KEGG, and Reactome with all specific thresholds *P* < 0.05 are shown in supplementary Tables 2, 3, and 4, respectively.
